# Functional disruption of macrophage migration inhibitory factor (MIF) suppresses proliferation of human H460 lung cancer cells by caspase-dependent apoptosis

**DOI:** 10.1186/1475-2867-13-28

**Published:** 2013-03-24

**Authors:** Yubiao Guo, Junna Hou, Yifeng Luo, Dujuan Wang

**Affiliations:** 1 Department of Pulmonary Medicine, the First Affiliated Hospital of Sun Yat-Sen University, Guangzhou 510080, China; 2 Department of Physiopathology, Zhongshan School of Medicine, Sun Yat-Sen University, Guangzhou 510080, China

**Keywords:** Macrophage migration inhibitory factor (MIF), Non-small cell lung cancer, Proliferation, Apoptosis, Caspase-3, Caspase-4

## Abstract

**Background:**

Macrophage migration inhibitory factor (MIF) is important in regulating cell proliferation and apoptosis in both normal and cancerous cells, and may be important in cancer progression and metastasis. In human non-small cell lung cancer (NSCLC), the underlying mechanisms responsible for MIF-dependent regulation of cellular proliferation, and cell death remain poorly appreciated.

**Methods:**

The human H460 lung cancer cell-line was treated with an optimally determined dose of 50 pmol/ml MIF siRNA, following which cell proliferation, cell cycle and apoptosis were analyzed. Additionally, known pathways of apoptosis including expression of Annexin-V, enhanced production of caspases-3 and −4 and expression of the Akt signaling protein were assessed in an attempt to provide insights into the signaling pathways involved in apoptosis following disruption of MIF expression.

**Results:**

Specific siRNA sequences markedly decreased MIF expression in H460 cells by 2 to 5-fold as compared with the negative control. Moreover, MIF miRNA dampened not only cellular proliferation, but increased the frequency of apoptotic cells as assessed by cell-surface Annexin-V expression. Entry of cells into apoptosis was partly dependent on enhanced production of caspases −3 and −4 while not affecting the expression of either caspase-8 or the Akt signaling pathway.

**Conclusions:**

In a model of NSCLC, knockdown of MIF mRNA expression dampened H460 proliferation by mechanisms partly dependent on entry of cells into apoptosis and enhanced production of caspase-3 and −4. MIF expression may thus be important in NSCLC progression. Targeting MIF may have clinical utility in the management of human lung cancer.

## Introduction

Lung cancer is the leading cause of cancer-related death in the world, and accounted for approximately 157,300 deaths in the United States in 2010 [1]. It is estimated that 85-90% of lung cancers are of the non-small-cell lung cancer type (NSCLC). With current therapies lacking adequate specificity and efficacy, the median overall survival rate of patients with metastatic NSCLC remains at approximately 1 year [2,3]. Moreover, It is clear that chemo-therapy has reached a plateau of activity in the treatment of NSCLC [4]. Thus, novel treatment strategies for targeting human lung cancer are urgently warranted.

Macrophage migration inhibitory factor (MIF) is considered a multifunctional cytokine secreted by a variety of cells such as macrophages [5], lymphocytes [6], eosinophils [7], epithelial cells [8], and endothelial cells [9], which importantly, is over-expressed in many different lung cancers [10-12]. In human lung adenocarcinoma, MIF modulates tumor cell migration and invasion, in part by inducing the activation of the Rho GTPase Rac, and membrane lipid raft stabilization; features that are important in both driving and sustaining tumor cell invasion [13-15].

The functions of MIF are predominantly immunoregulatory, serving important roles in inflammation, cell-mediated and innate immunity [16-19]. In addition, MIF displays a dominant role in diseases that are characterized by pro-inflammatory pathways, such as the severity of rheumatoid arthritis [20], cardiac dysfunction that is seen in sepsis [21], Crohn’s disease [22], and many different cancers [23-25].

Since the discovery of MIF almost 50 years ago, more recent work has identified MIF as a key factor in the development and progression of human cancers, and particularly in the metastatic potential of colorectal and lung tumors [13,23-29]. Given the known functions of MIF in pro-inflammatory pathways, and the weight of evidence associating inflammatory pathways and the development of cancer, it comes as no surprise that MIF is emerging as a key player in the progression and growth of many tumors [23-29]. It is thought that the ability of MIF to suppress the anti-inflammatory effects of glucocorticoids is central to the inflammatory promoting functions of MIF, for example in diseases such as acute respiratory distress syndrome [30,31].

The ability of MIF to suppress anti-inflammatory pathways is highly relevant to the growing appreciation of chronic inflammatory pathways promoting tumor growth and metastatic development. In host immune defense, inflammatory cytokines and other inflammatory mediators assist in the clearance of infection, eradication of tumors and in the repair or maintenance of intact tissues and organs. However, the inflammatory milieu, and particularly in chronic inflammation, provides an environment that assists tumor development and metastasis. The biological activities of MIF are thought to contribute to these processes by inhibiting the regulatory functions of p53 and thus blocking apoptosis (programmed cell death). It is thought that this is achieved by certain tumors sustaining the activation of the ERK signaling pathway via the functional activation of MIF. Such conditions not only attenuate cell death, they also promote tumor cell invasion and induce the expression of COX-2 and PGE-2 which collectively induce tumor cell growth, tumor cell survival and metastasis. These are conditions that favor de novo angiogenesis, thus providing a blood supply to metastatic tumors [16,32-34].

In the specific setting of non-small cell lung cancer (NSCLC), monocyte-derived macrophage secretion of MIF is augmented by NSCLC cells, and secretion of MIF may contribute to local angiogenic activity and tumor metastasis in cell culture models and mouse models of tumor development [11,35-37]. A major breakthrough in our understanding of the role of MIF in tumor metastasis in NSCLC was the identification of CD74 (the invariant chain of the HLA class II peptide) as the cell surface receptor for binding MIF [10,38]. Although very little is known of the relevance of CD74 in many lung cancers, the expression of CD74 in gastric carcinoma has been associated with a poor prognosis [39,40]. More recently, association of CD74 and MIF co-expression in lung cancers [10], and the identification of MIF by label-free proteomic approaches as one of many promising biomarkers in NSCLC [41], provides additional evidence of the importance of MIF in lung cancer development and progression.

Thus, in the current study, we set out to employ H460 cells as a relevant model system to explore the functional role of MIF in NSCLC. Further, we wished to assess the molecular mechanisms responsible for the anti-tumor effects of functionally dampening MIF expression using specific siRNA sequences. We found that MIF siRNA transfection inhibited both the proliferation and induced the apoptosis of H460 cells through mechanisms that were dependent on enhanced production of caspase-3 and caspase–4, sustained expression of the Akt/protein kinase B (phosphoinositide 3-kinase, PI3K) signaling pathway, proliferation arrest and promotion of an apoptotic mode of programmed cell death.

## Materials and methods

### Cell culture maintenance and transfection

The human non-small cell lung cancer cell-lines H460 and A549, were both obtained from the Cell Bank of the Animal Experiment Center, North School Region, Sun Yat Sen University, Peoples Republic of China. H460 cells were cultured in RPMI-1640 medium (GIBCO, USA) and A549 cells were cultured in DMEM medium (GIBCO, USA) where both cultures were supplemented with 10% newborn calf serum. Both cell-lines were maintained in a fully humidified incubator at 37°C and an atmosphere of 5% CO_2_ in air.

For transfection experiments, two independent siRNA species were designed to target knockdown of functional MIF expression and were obtained from Invitrogen (San Diego, CA, USA). The sequences of each miRNA species were:

1) Sense 5^′^-AUAGUUGAUGUAGACCCUGUCCGGG-3^′^ Antisense5^′^-CCCGGACAGGGUCUACAUCAACUAU-3

2) Sense 5^′^-UUGGUGUUUACGAUGAACAUCGGCA-3^′^ Antisense5^′^-UGCCGAUGUUCAUCGUAAACACCAA-3

The negative control siRNA was also obtained from Invitrogen, and had the following sequence:

Sense 5^′^-GCGCGCUUUGUAGGAUUCGdTdT −3^′^, Antisense 5^′^ -CGAAUCCUACAAAGCGCGCdTdT −3.

Cell-lines at an exponential phase of proliferation were seeded into 6-well culture plates, 1.5 × 10^5^ cells per well. When the confluence of the cells approximated 30-40% of the available surface area of the culture wells, the cells were transfected with 50 pmol/ml siRNA (a dose that was found to be optimal in dose-dependent experiments), using lipofectamine 2000 reagent (Invitrogen, USA) following the manufacture’s protocol. Cells transfected with the negative control siRNA were used to control for the specificity of the miRNA MIF knockdown studies. The transfection efficiency was monitored by observation of the frequency of immunofluorescent positive cells by microscopic examination.

### Determination of cell proliferation by MTT assay

Approximately 5 × 10^3^ cells per well were seeded into 96-well plates and transfected with MIF siRNA or negative control siRNA, both at a dose of 50 pmol/ml using lipofectamine 2000 reagent (Invitrogen, USA) following the manufacture’s protocol. At the indicated time points of 24 h, 48 h and 72 h post-transfection, the extent of cellular proliferation was measured by MTT assay. This was done by adding MTT reagent (20 μl) to each well and incubating the plates for an additional 4 h at 37°C. At the conclusion of the assay, the medium was aspirated, and dimethyl sulfoxide (150 μl) was added to each well to dissolve the formazan product following metabolism of the MTT reagent. Absorbance values of the formazan product were measured at a wavelength of 490 nm (A_490_). Each experiment was repeated at least three times.

### Plate cloning assay

A plate-cloning assay was also carried out 24 h after the transfection of MIF siRNA, In this assay, H460 cells were collected, trypsinized, and plated into 6-well plates of 200 cells per well. Cells were cultured in complete medium (2 ml/well) continuously for 10 days, following which they were fixed, stained, and observed for the formation of visible cultured cell clones by light microscopy. Aggregation of ≥50 cells was considered as a clone. The percentage of clone formation was calculated according to the following equation:

$$\begin{array}{l} \mathrm{Percentage}\;\mathrm{of}\;\mathrm{clone}\;\mathrm{formation}\\ \;=\;\left(\mathrm{clone}\;\mathrm{number}/\mathrm{plated}\;\mathrm{cell}\;\mathrm{number}\right)\;\times \;100\% \end{array}$$

### Flow cytometric determination of H460 Apoptosis

The H460 cell-line was seeded at a density of 1.5 × 10^5^ cells/well into 6-well culture plates under conditions described above. Cells were treated with either MIF siRNA or NC siRNA (for the control group) for a period of 48 h of continuous culture in the presence of these siRNA species. At the conclusion of the assay, cells were harvested by trypsinization, washed three times in PBS and resuspended in 0.5 ml PBS. Immediately after resuspension of the cells, propidium iodide (PI) and a FITC-conjugated monoclonal antibody specific for Annexin V (KaiGi Technology, Guangzhou, China) were incubated with the cells at 4°C for a period of 30 minutes. Cell apoptosis was measured using Flow cytometry (Becton Dickinson Biosciences, Inc., NJ, USA).

### Western immunoblotting

Treated H460 cells were lysed in a lysis buffer supplemented with a protease inhibitor cocktail (Tissue or Cell Total Protein Extraction Kit, Shanghai, China). After 10 min incubation on ice, the cell suspension was centrifuged at 12000 g for 20 min at 4°C. Soluble protein fractions were then analyzed by Western immunoblotting performed as follows: First, protein samples were resolved on 12-15% SDS-PAGE gels. The protein bands were transferred onto PVDF membranes (Millipore, USA) which were then blocked overnight in TBS-Tween 20 (TBST) buffer containing 5% w/v skimmed milk proteins. The membranes were washed three times with TBST for 10 min each. Second, the membranes were incubated with an appropriate dilution of a specific primary antibody targeted against MIF (Abcam, USA), caspases-3, -4 and −8 and AKT (all obtained from Cell Signaling Technology, USA) with gentle shaking overnight at 4°C. Thirdly, the membranes were washed thoroughly with TBST and incubated with a HRP-conjugated secondary antibody (Cell Signaling Technology, USA) for 1 h at room temperature. Finally, after the membranes were washed with TBST, the resolved and transferred protein signals were detected by enhanced chemiluminescence (ECL). The stained bands were scanned and the relative optical densities measured for semi-quantitation of the relative expression levels of each ECL detected protein band.

### Statistical analysis

The results were expressed as the arithmetic mean ± one standard deviation (SD) about the mean. All data were the product of at least three independent experiments. The data were analyzed by one-way analysis of variance (ANOVA) using SPSS 16.0 statistical analysis software (SPSS Inc, Chicago, IL). An alpha value of *P* < 0.05 was considered statistically significant.

## Results

### MIF is expressed in H460 and A549 cells and siRNA-mediated MIF knockdown in H460 cells

**Figure 1:**
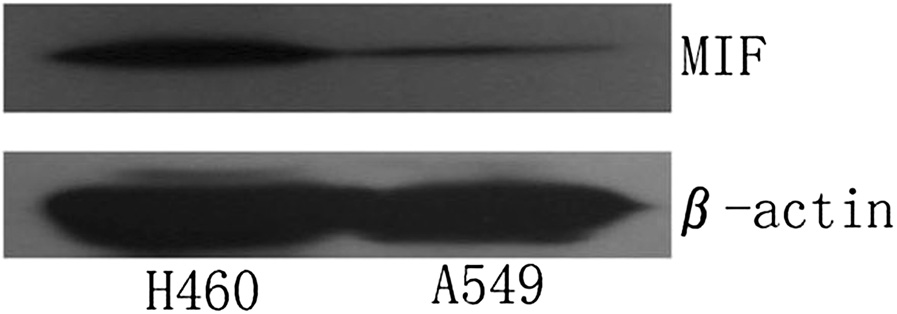
**Expression of MIF protein by Western immunoblot analysis in H460 and A549 cells.** A549 cells showed a weaker signal for MIF expression as compared with H460 cells normalized to expression of β-actin. For this reason, we selected the H460 cell-line as a model of human non-small cell lung cancer in all subsequent experiments.

**Figure 2:**
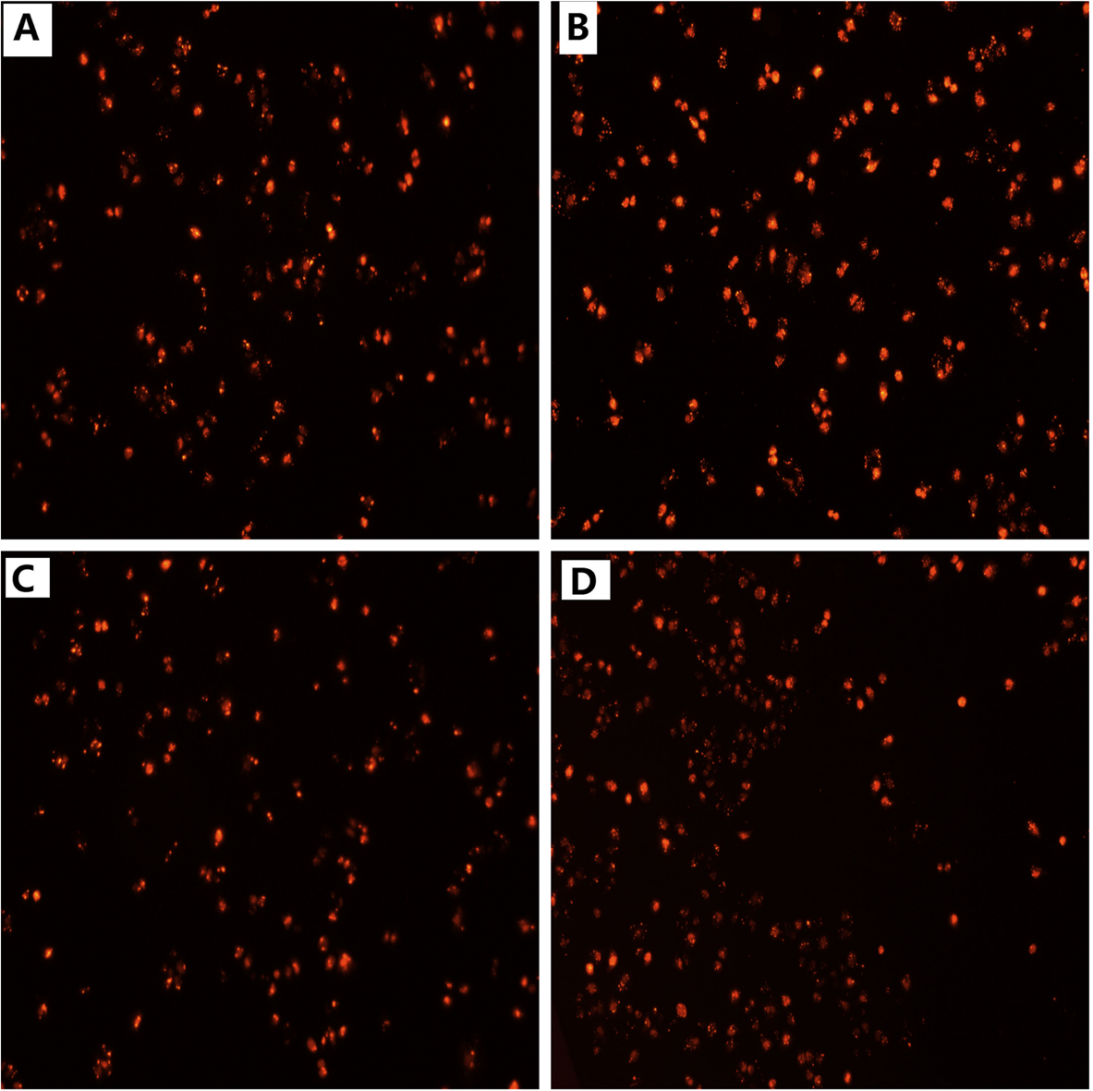
**Dose-dependent detection of siRNA transfection efficacy by immunofluorescence observation.** The following doses of siRNA were assessed: **A**: 30 pmol/ml. **B**: 50 pmol/ml. **C**: 70 pmol/ml. **D**: 100 pmol/ml. By visual inspection of the fluorescence intensity and frequency of cells transfected, we selected a dose of 50 pmol/ml in subsequent experiments.

Prior studies have reported that MIF is over-expressed in human lung adenocarcinomas [12,13,29]. We evaluated the expression of MIF in H460 and A549 cells, and found that the expression of MIF was highest in H460 cells (Figure 1). In pilot experiments, we found that a concentration of 50 pmol/ml of specific MIF siRNA was optimal for disrupted expression of MIF (see below). The transfection efficiency was monitored by immunofluorescence observation and we found that the transfection efficiency was more than 70% (Figure 2).

In addition, we found that at siRNA concentrations greater than 50 pmol/ml, the extent of cell death increased dose-dependently with increasing siRNA concentrations. Therefore, we selected a dose of 50 pmol/ml for optimal transfection of H460 cells with siRNA for all the following experiments.

**Figure 3:**
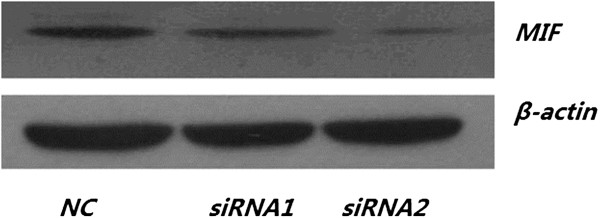
**siRNA-mediated knockdown of MIF expression in H460 cells detected by Western blot.** In MIF siRNA-transfected H460 cells (**A**), we observed a an approximately two (siRNA 1) to five (siRNA2) fold weaker signal of MIF protein expression as compared with the negative control (NC) group normalized to the expression of β-actin (**B**).

After incubating the cells for 48 h with the MIF siRNA species, we determined the relative expression of MIF knockdown by Western immunoblotting (Figure 3). We found that the expression of MIF protein was significantly reduced in the cells transfected with MIF siRNAs as compared those cells treated with the NC siRNA. These observations suggested that MIF siRNAs could dampen the functional expression of MIF in H460 cells very efficiently (Figure 3).

### Determination of the effects of MIF siRNA on H460 cellular proliferation

**Figure 4:**
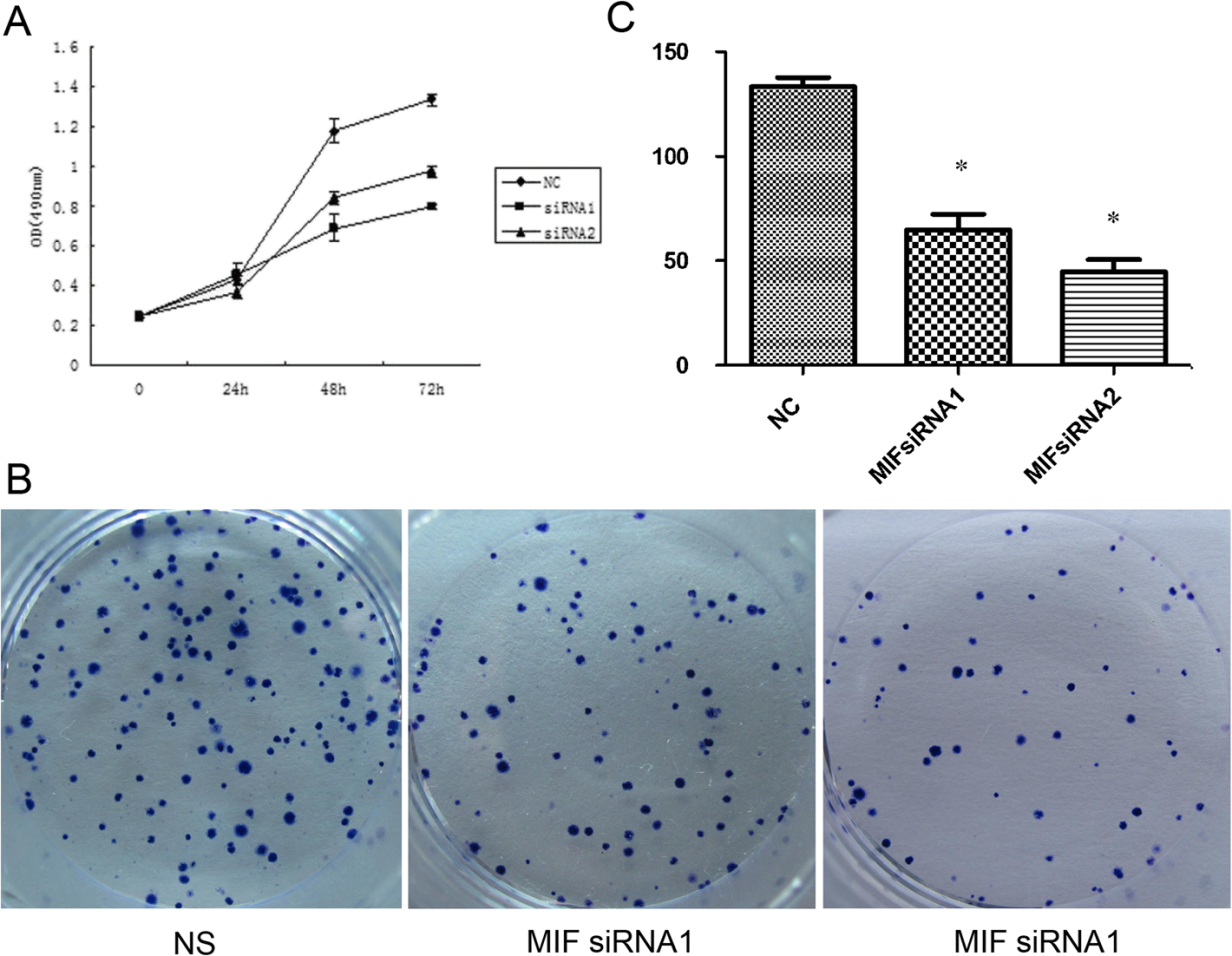
**Effect of MIF siRNA on the proliferation of H460 cells. (A)**; MTT assay showed that treatment of H460 cells with MIF siRNA inhibited their proliferation. Each point in the curve represents the arithmetic mean OD values ± SD from representative experiments that were performed in triplicate. (**B**); Plate cloning assay indicated that MIF siRNA inhibited the proliferation of H460 cells. Image (**C**); Quantification of the data obtained from the plate cloning experiments. Data were expressed as arithmetic mean ± SD, Indicated levels of statistical significance were; *: *P* < 0.05 as compared with the NC Group. NS: Negative control group.

Using the MTT metabolic and viability assay, the cellular proliferation and viability of treated H460 was determined (Figure 4A). We found that in H460 cells treated with MIF siRNA displayed significantly reduced proliferation as compared with cells treated with the NC siRNA control sequence (p <0.05). In addition, the plate cloning assay indicated that MIF siRNA inhibited the proliferation of H460 cells (Figure 4B) which was concordant with the data obtained by MTT assay.

**Figure 5:**
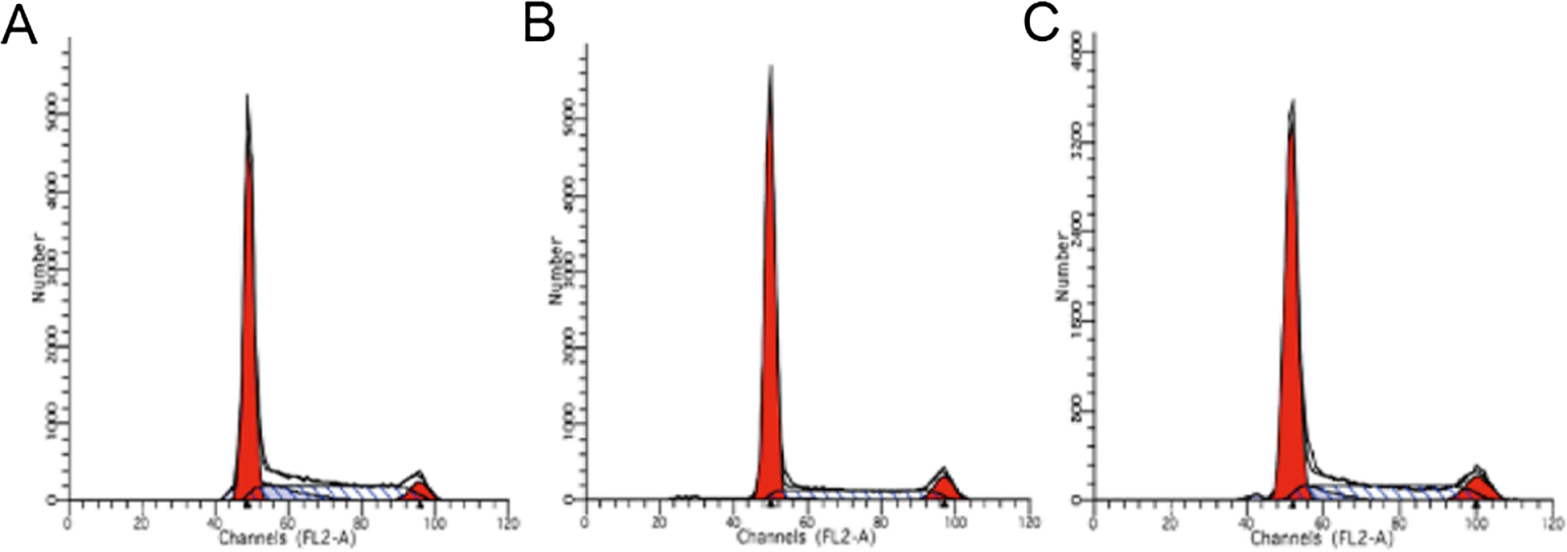
**Effect of MIF siRNA on the cell cycle of H460 cells.** In propidium iodide stained cells, the cell cycle phases were determined in H460 cells by flow cytometry. The data is described for the cell cycle of H460 cells in the NC group **(A)**, following treatment with MIF siRNA1 **(B)**, and after treatment with MIF siRNA2 (**C**).

We also assessed the cell cycle dynamics (Figure 5) of H460 cells treated with the NC siRNA control (A), and following treatment with either MIF siRNA1 (B), or MIF siRNA2 (C). In these cell cycle analyses, we could not determine any significant effect on the major cell cycle compartments wherein the G0/G1 peaks, S-phase and G2/M phases of the cell cycle appeared quite similar (Figure 5). Nonetheless, collectively, these data suggest an important functional role for MIF in lung tumor cell proliferation.

### Knockdown of functional MIF expression promotes apoptosis

**Figure 6:**
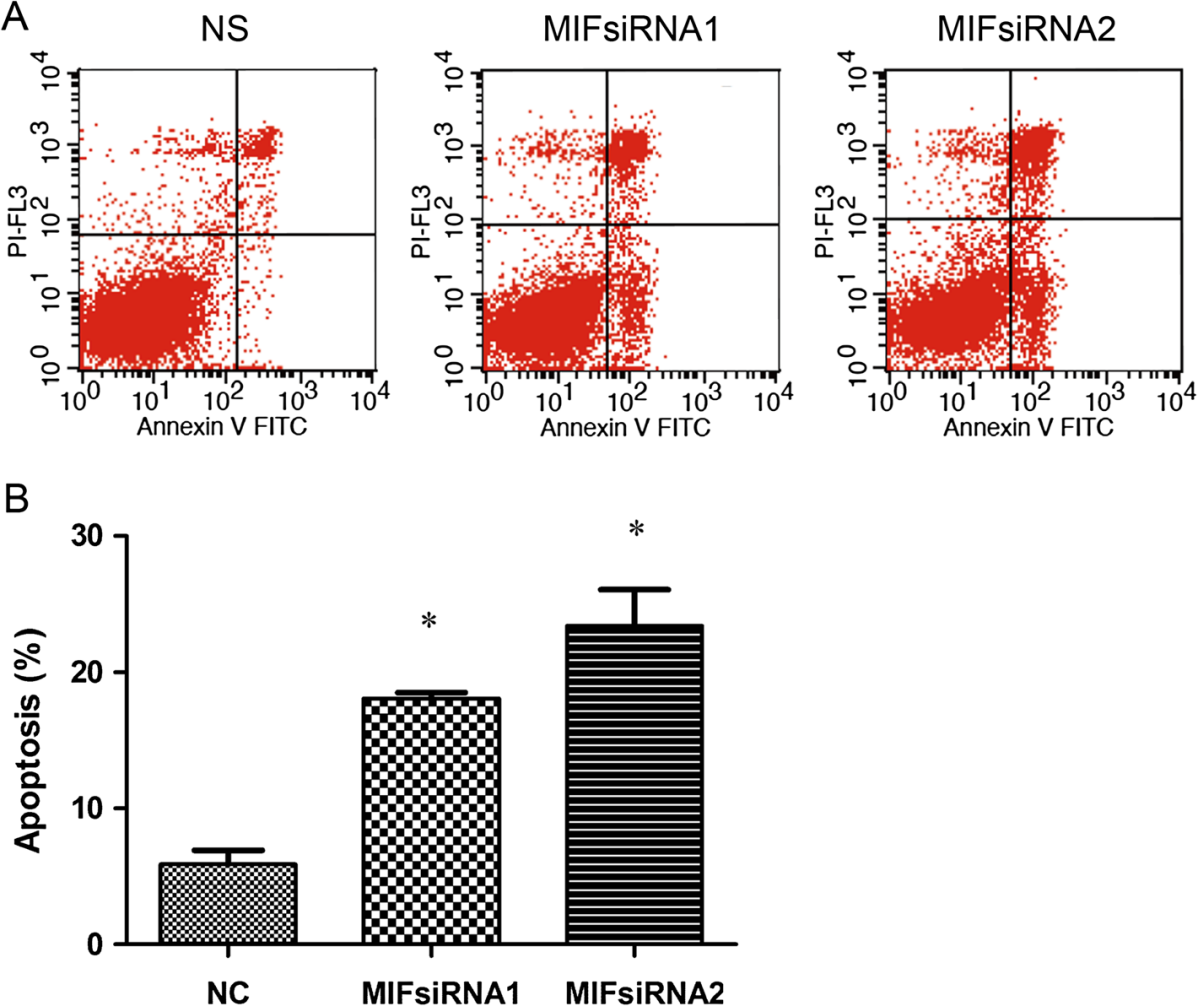
**Flow cytometric determination of the effect of MIF siRNA on Apoptosis of H460 cells.** (**A**); Cultured H460 cells were divided into three groups: A: cells transfected with NC siRNA, (**B**) and (**C**); cells transfected with MIF siRNA (siRNA1 or siRNA2 respectively). After a 48 h treatment, the cells were harvested for quantitation of apoptosis by determining changes in the cell surface expression of Annexin-V. (B) shows a description of the observed frequency of cells undergoing apoptosis which were found to be much higher in the MIF siRNA1 (18.09 ± 0.41%) and MIF siRNA2 (23.38 ± 2.67%) treated groups than in the negative control (NC) treated group (5.87 ± 1.05 and p < 0.05) of H460 cells respectively.

Consistent with the observations indicating apoptotic modes of cell death in H460 cells treated with MIF siRNA (Figure 5), we next pursued multi-parameter flow cytometric analysis of siRNA transfected H460 cells to obtain more sensitive and quantitative details of a possible apoptotic mode of cell death (Figure 6A and B). Following 48 h of culture of MIF siRNA transfected H460 cells, flow cytometry was used to quantify the expression of Annexin-V in the absence of PI staining following MIF siRNA treatment (Figure 6A), a condition which indicated apoptosis. We found that in cells treated with both MIF siRNA1 and MIF siRNA2, displayed significantly higher levels of Annexin-V staining (18.09 ± 0.41% and 23.38 ± 2.67% respectively) as compared their negative control siRNA treated counterparts (5.87 ± 1.05%, p < 0.05, Figure 6A and 6B). These observations collectively suggested that MIF may serve important regulatory roles in apoptosis.

### Pathway analysis by western immunoblotting

**Figure 7:**
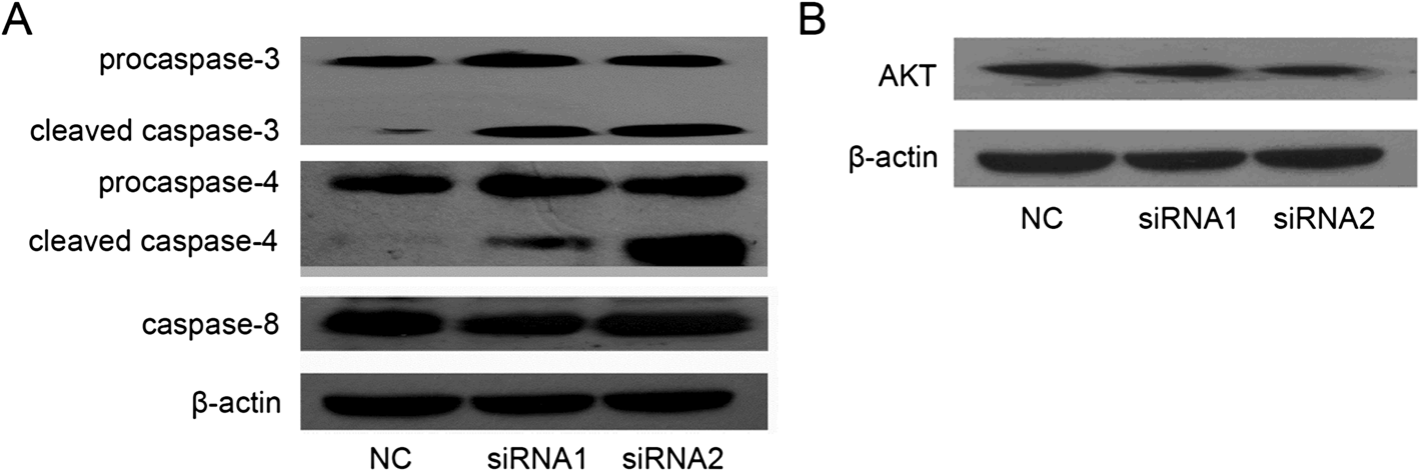
**Effects of MIF siRNA on cellular expression of caspase-3, caspase-4, caspase-8 and Akt in H460 cells.** To determine the expression of caspase-3, caspase-4, caspase-8 (**A**) and Akt (**B**) in differentially siRNA-treated H460 cells, cultures were harvested after a 48 h treatment, and protein expression patterns assessed by Western immunoblotting analysis and ECL with an appropriate dilution of specific primary antibodies. Beta-actin was used as loading control for all proteins studied.

In an attempt to elucidate the putative mechanisms responsible for the possible anti-tumor effect of MIF siRNA, we assessed the relative expression levels of proteins thought to be associated with apoptosis and cellular proliferation. We found that the expression of the cleaved band (i.e. caspase-3 and −4) of pro-caspase-3 and −4 were significantly higher in the MIF siRNA groups (Figure 7A), while the expression of both caspase-8 (Figure 7A) and Akt in H460 cells remained unaltered (Figure 7B) irrespective of the siRNA treatment. Collectively, these observations suggest that MIF siRNA not only blocks cell proliferation of H460 cells, but also promotes an apoptotic mode of programmed cell death that may be dependent, at least in part, on enhanced cleavage of pro-caspases-3 and −4 to their mature functional procaspase counterparts (i.e. caspase-3 and −4) (Figure 7A).

## Discussion

Several reports in the literature have indicates the critical role of MIF as a regulator of innate and adaptive immunity, inflammation and tumor progression [16,42]. Increased expression of MIF has been reported in hepatocellular carcinoma, prostate carcinoma, lung adenocarcinoma, neuroblastoma and colorectal cancers [13,24,25,27,29]. High expression of MIF in lung cancer patients predicts a worse prognosis for disease free and overall survival [11,43]. It has been shown that CD74 was the cell surface receptor for MIF [10,38], and that MIF promotes sustained ERK/MAPK activation through occupation of the cell surface CD74 receptor [44,45].

A number of reports have shown that MIF is capable of blocking p53-dependent apoptosis [46], and can activate the PI3K/Akt pathway [47,48], as well as promoting endothelial cell proliferation and differentiation [49-51]. These findings confirm that MIF plays an important role in the development and promotion of human malignancies. Since many of the mechanisms responsible for the multifactorial functions of MIF have still to be identified, we have provided important new information with regard to the role of MIF in regulating tumor cell proliferation and programmed cell death by caspase-3 and caspase-4 dependent pathways.

In our study, knockdown of the functional expression of MIF markedly decreased H460 cell proliferation and induced apoptosis as seen by augmented expression of Annexin-V following treatment of H40 cells by MIF siRNA. Additionally, caspases plays an essential role in cell apoptosis and indeed most cell-inducing stimuli direct apoptosis through the activation of a specific sequence of caspase proteins. Caspases are cysteine proteases, and functionally related to interleukin-1β converting enzyme (ICE). Activation of ICE-like proteases by stimuli that trigger apoptosis, act on substrates such as poly(ADP-ribose) polymerase or PARP, and activate other enzymes such as endonucleases and transglutaminase, which leads to energy (ATP and ADP) depletion, ER stress, protease activation, cytoskeletal disorganization, and apoptotic body formation. Thus, the enhanced cleavage of pro-caspase-3 and pro-caspase-4 to their biologically active pro-caspase counterparts that we found in this study following treatment of H40 cells with MIF siRNA, is a key feature of the highly regulated cell death process of apoptosis.

Significant evidence has accumulated suggesting that the endoplasmic reticulum (ER) plays a crucial role in the execution of apoptosis [52,53]. For example, caspase-12, has been shown to induce apoptosis in response to ER stress [54] and in humans, the ER-mediated killing role of caspase-12 has been found to be substituted by caspase-4 [54,55]. Moreover, caspase-4 has been found to be a specific mediator of ER stress and may play an important role in the ER-stress pathway [55,56].

By contrast, caspase 3 is downstream effector protein, and it takes part in the functionally crucial decision and execution phases of the apoptosis process [57]. Caspase-8 is generally considered to be an initiator caspase due to its ability to be associated with the cell surface death receptor that transduces apoptosis via the structural signaling complexes FADD/MORT1 and RAIDD/TRADD. Indeed, the amino terminal of the death effector domain (DED) of FADD/MORT1 is required for death induction and interacts structurally with the prodomain of caspase-8, which recruits the FLICE/MACH death effector proteins that implement apoptosis.

In our study, the expression of the cleaved band of both caspase-3 and caspase-4 are significantly increased in the MIF siRNA groups. This data not only suggests that siRNA-mediated knockdown of MIF can promote cellular apoptosis in human lung H460 cells, but does so in caspase-3 and caspase-4 dependent mechanism. It is tempting to speculate that the ER-stress pathway is involved in this process following pro-caspase cleavage and activation, yet this will need to await formal demonstration in our model system described in the current work.

In conclusion, we have shown that MIF increases the proliferation and blocks, at least in part, the apoptosis of H460 cells via caspase-3 and caspase-4 dependent pathways under conditions where the functional expression or activity of both caspase-8 and the Akt signaling pathway remains unaltered. We propose that dampening of the functional expression of MIF could reasonably be exploited as a clinically useful strategy in the management of many tumors including lung cancer.

## Abbreviations

MIF: Macrophage migration inhibitory factor; siRNA: Small interfering RNA; ER-stress: Endoplasmic Reticulum Stress; PI: Propidium iodide; PI3K: Phosphoinositide 3-kinase; AKt/PKB: Protein Kinase

## Competing interests

The authors declare that there are no conflicts of interests.

## Authors’ contributions

YBG: Conceived and designed the experiments; JNH, DJW: Performed the experiments and analyzed the data; YFL, HLY: Contributed reagents/materials. All authors read an approved the final draft.
