# Correction: Functional disruption of macrophage migration inhibitory factor (MIF) suppresses proliferation of human h460 lung cancer cells by caspase-dependent apoptosis

**DOI:** 10.1186/1475-2867-13-84

**Published:** 2013-08-20

**Authors:** Yubiao Guo, Junna Hou, Yifeng Luo, Dujuan Wang

**Affiliations:** 1 Department of Pulmonary Medicine, the First Affiliated Hospital of Sun Yat-Sen University, Guangzhou 510080, China; 2 Department of Physiopathology, Zhongshan School of Medicine, Sun Yat-Sen University, Guangzhou 510080, China

## Correction

After publication of the original article [1] it came to the authors attention that an incomplete version of Figure three (Figure 1 here) was published with the article. The complete figure and new figure legend are presented in this correction article.

**Figure 1:**
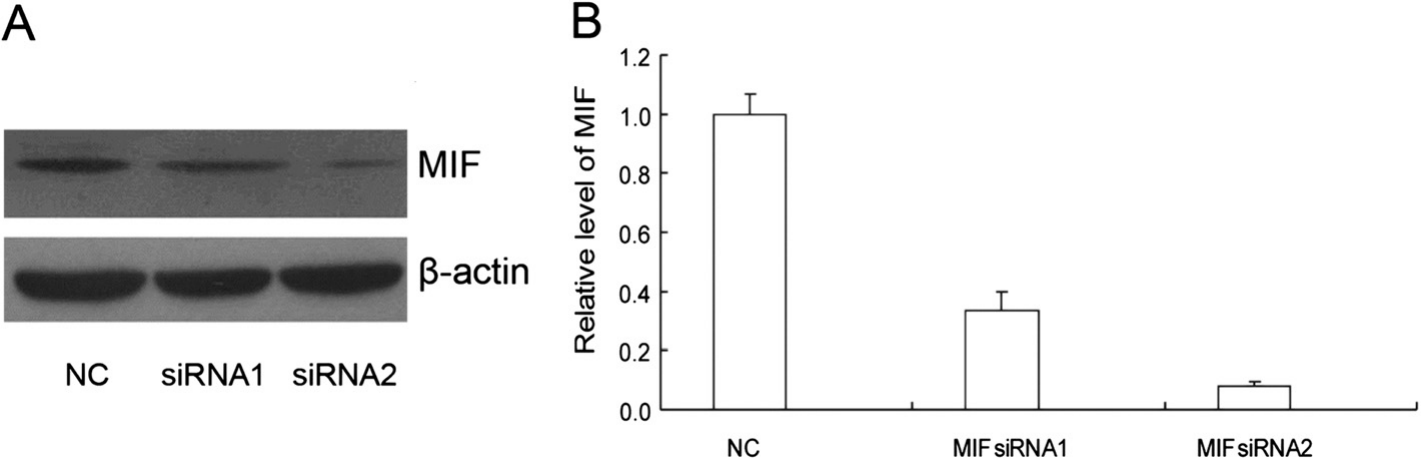
**siRNA-mediated knockdown of MIF expression in H460 cells detected by Western blot. (A)** is a western blot of MIF expression in H460 cells treated with NC and two MIF siRNAs for 48 hours. In MIF siRNA-transfected H460 cells, we observed an approximately two (siRNA 1) to five (siRNA2) fold weaker signal of MIF protein expression compared with the negative control (NC) group normalized to the expression of β-actin. **(B)** is a densitometric analysis of the western blot. This shows the relative densities of protein levels which were measured by Quantity One (Bio-Rad company) software.
